# Nanoremediation of As and metals polluted soils by means of graphene oxide nanoparticles

**DOI:** 10.1038/s41598-020-58852-4

**Published:** 2020-02-05

**Authors:** Diego Baragaño, Rubén Forján, Lorena Welte, José Luis R. Gallego

**Affiliations:** 1 INDUROT and Environmental Technology, Biotechnology and Geochemistry Group, Campus de Mieres, Universidad de Oviedo, Mieres, Asturias Spain; 2 Kleinscale S.L., Calle Montoro 4 9D, 28922 Alcorcón, Madrid Spain

**Keywords:** Environmental, health and safety issues, Pollution remediation

## Abstract

The capacity of graphene oxide nanoparticles (nGOx) to reduce or increase As and metals availability in polluted soils was compared with that of zero valent iron nanoparticles (nZVI). The nanomaterials used in this study were characterized by X-ray techniques, CHNS-O analysis, dynamic light scattering, and microscopy procedures such as atomic force microscopy. To assess the capacity of these materials to immobilize pollutants, field samples of two soils were treated with nZVI and nGOx at a range of doses (0.2%, 1% and 5%). Availability tests were then performed. nGOx effectively immobilized Cu, Pb and Cd, but mobilized As and P (even at low doses), in the latter case irrespective of the simultaneous presence of high concentrations of metals. In turn, nZVI promoted notable immobilization results for As and Pb, a poorer result for Cd, and an increased availability for Cu. Soil pH and EC have been slightly affected by nGOx. On the whole, nGOx emerges as a promising option for mobilization/immobilization strategies for soil nanoremediation when combined with other techniques such as phytoremediation.

## Introduction

Industrial activities have released large amounts of metals (Cu, Cd, Pb, Zn, etc.) and metalloids (especially As) into the environment and caused serious damage to the ecosystem^1^. In addition, metals and metalloids can endanger human health, as many of them are toxic even at very low concentrations, and even carcinogenic (e.g. As) and mutagenic^2^. Unlike organic pollutants that can degrade into harmless small molecules, the abovementioned inorganic pollutants are recalcitrant to many biochemical reactions and thus particularly difficult to remove from soils^3^.

Regarding As, several studies have confirmed that between 52,000 and 112,000 tons of this metalloid are released into the environment every year^1,4,5^. Many diseases, such as lung cancer, skin cancer, bladder cancer, etc., can be caused by contact with As^6,7^. Given that As is highly toxic for humans, and also for plants and animals, it has been classified as a priority hazardous pollutant. Unlike the aforementioned metals, As is an anionic contaminant. In this regard, simultaneous remediation of soils affected by anionic and cationic contaminants is a challenge^8^; i.e., As remediation is even more complex in contexts of concurrent pollution with metals. In fact, classical remediation approaches attempt, among other objectives, to raise the pH of soils contaminated with cationic metals and thus stabilize them. However, this approach causes As to be solubilized^9^. Overall, the various remediation strategies available focus on the disjuncture between immobilization and mobilization of the pollutants^10^.

Soil nanoremediation by means of Fe-nanoparticles (nZVI) simultaneously reduces the availability of As and metals in polluted soils, as shown in recent pilot-scale *in situ* trials^11^, and by means of hybrid soil-washing techniques^12^. nZVI have a Fe° core surrounded by an oxide/hydroxide shell, which grows thicker as iron oxidation progresses. In addition, nZVI present a large specific surface area, thus offering a high adsorption capacity and high reactivity^13,14^. The greater reactivity often ascribed to nanoparticles is the result of a larger overall surface area, greater density of reactive sites on the particle surfaces, and/or higher intrinsic reactivity of the surface sites^15^. Metal-nZVI interactions can be summarized as the following: reduction, adsorption, oxidation/reoxidation, co-precipitation and precipitation^16^. Other nanocompounds such as Fe-oxides^17,18^ have also been tested as an alternative to nZVI, although most studies have focused on water remediation^19,20^. In this context, graphene oxide nanoparticles (nGOx) have also been used in the aquatic environment to remove metals and other toxic elements^6,21^. However, to the best of our knowledge, the capacity of nGOx for soil remediation purposes has not been tested to date.

Graphene oxide is a novel extremely oxidative form of graphene acquired by graphite chemical exfoliation^22^, and it is considered one of the most interesting materials to have emerged in recent years^23^. The reactivity of nGOx is conferred by a large specific surface area and the presence of a large amount of chemically bonded oxygen on the surface, including carbonyl and carboxyl groups at the layer ends and hydroxyl and epoxy groups on the base plane^24–27^. Thus, the functional groups containing oxygen atoms have lone electron pairs that can efficiently bind a metal ion to form a metal complex via electrostatic interaction and coordination^23,28^. Also, the presence of these oxygen atoms allows hydrogen to bind to water molecules, thereby conferring nGOx solubility in water^29^. This property can promote reactivity with the pollutants in the solution phase of soils.

Given the preceding considerations, the main objective of this study was to determine the effect of nGOx addition to soils in two distinct paradigmatic scenarios of As-polluted soils. To this end, nGOx was tested in a soil with high concentrations of As, Cd, Cu, Pb and Zn. Complementary experiments were also carried out soil from a brownfield affected only by a high concentration of As. In addition, in both cases, we compared the effects of nGOx with those of nZVI, the most widely used type of nanoparticle in soil remediation.

## Results

### Characterization of synthesized graphene oxide nanoparticles

**Figure 1:**
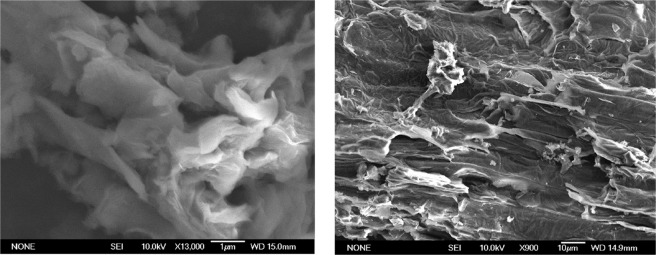
Morphology of nGOx as observed in SEM images.

**Figure 2:**
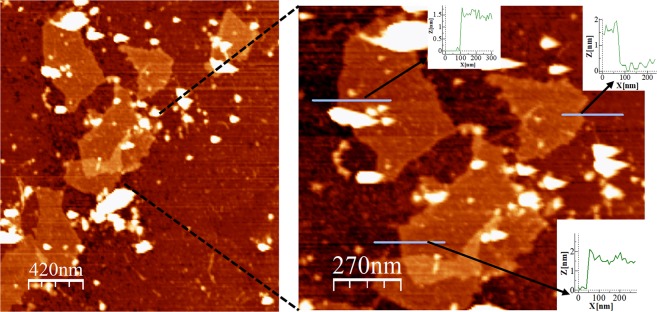
AFM images of nGOx and topographic profiles.

**Figure 3:**
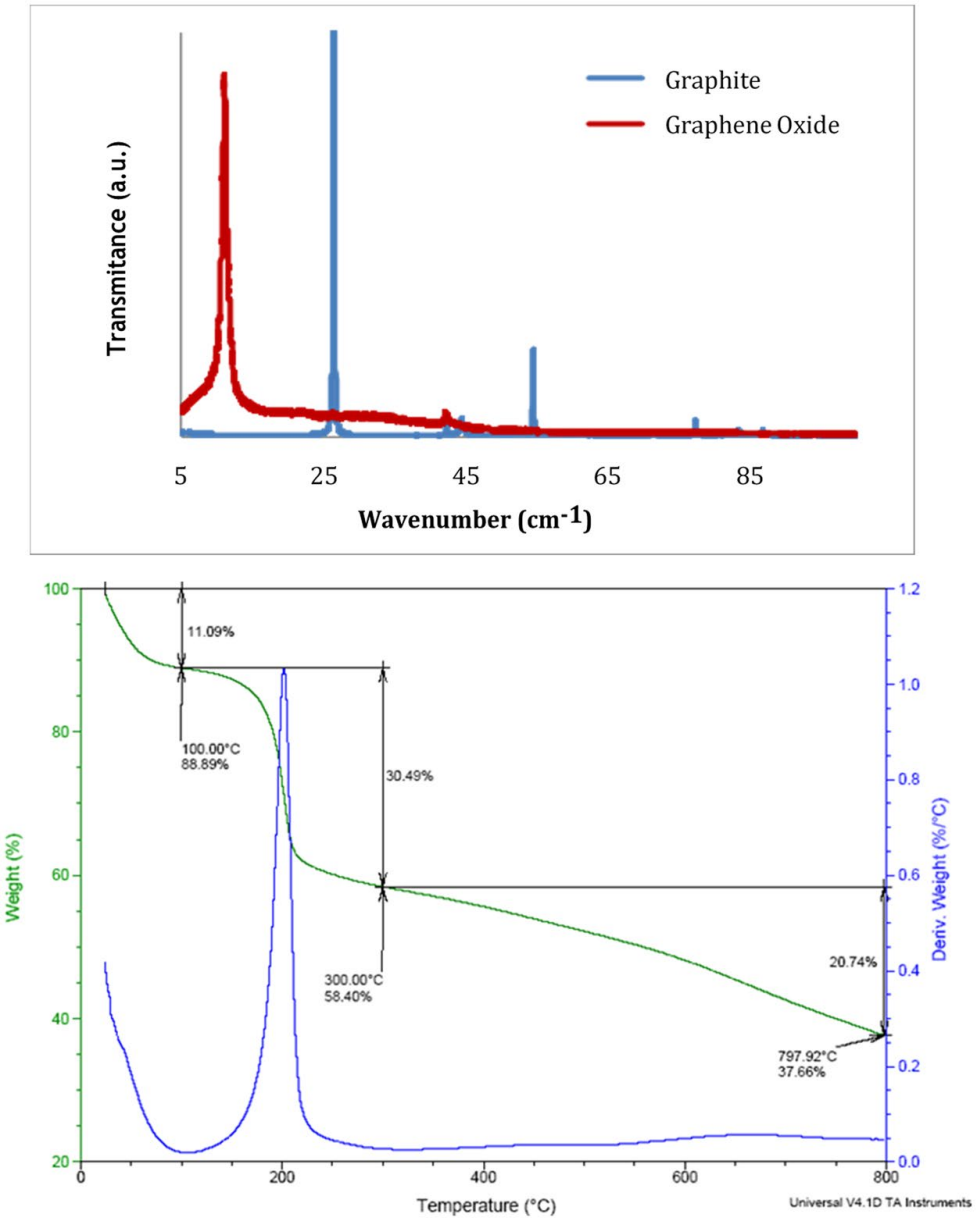
(**A**) XRD patterns of graphite and graphene oxide powders. (**B**) TGA analysis of nGOx.

SEM revealed the characteristic flake shape of nGOx (Fig. 1). The chemical analysis obtained by CHNS determination revealed the presence of 49.75% of C, 2.39% of H, 0.01% of N and 0.95% of S, and thus the oxygen content was estimated to be 46.9%. AFM analysis was used to determine flake thickness (Fig. 2). Several topographic profiles were measured, and an average thickness of 1.5 nm was determined, which is consistent with the results of other studies^30^. The XRD patterns of graphite and graphene oxide powders are shown in Fig. 3A. The diffraction peak of graphite at 26° corresponded to an interplanar spacing of (002) hexagonal layers of carbon atoms (d002 = 3.36A)^31^. However, this peak was absent in graphene oxide (GO), and another peak diffraction appeared at the range 9.0–11.20°, corresponding to (001) plane of GO^32^. The change in the XRD diffraction peak from 26° to 10° indicates the transformation of graphite into graphene oxide^33,34^.

The TGA plot consisted of three weight losses (Fig. 3B). First, a mass loss of approximately 11.9% occurred at 373 K, due to the elimination of absorbed water on the sample. Next, a loss of 30.5% was detected at 573 K, corresponding to the decarboxylation of graphene oxide. Finally, from 1073 K, successive decompositions of the material took place. These results are in agreement with Chung *et al*.^25^ and Sofla *et al*.^35^. The specific surface area determined through BET was 150 ± 2 m^2^.g^−1^ (n = 3). Similar results were obtained by other authors^25^.

The zeta potential of nZVI was −32.6 mV due to the polyacrylic acid (PAA) coating^36^. In contrast, nGOx are not covered, so the zeta potential value, −13 mV, corresponded to the functional groups of the material. The pH value of nGOx was 2.5 in the three prepared suspensions, which indicates that is an acidic material. In contrast, nZVI presented a basic pH value of 11.5.

### Influence of nZVI and nGOx on the available concentrations of Cu, As, Cd, Pb and Zn in the As-Metals polluted soil (AM)

**Figure 4:**
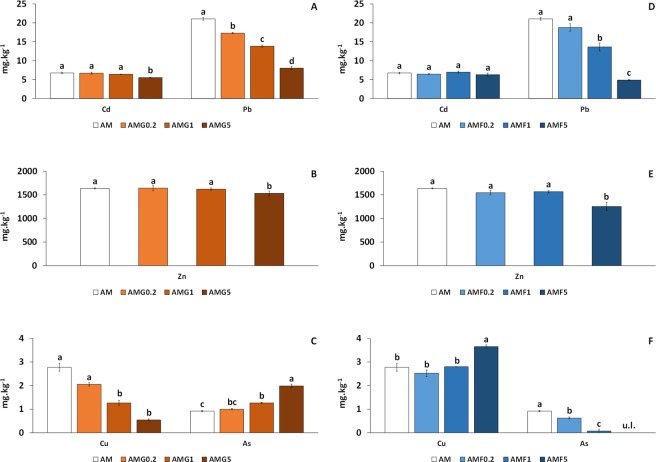
Influence of nGOX (**A**–**C**) and nZVI (**D**–**F**) on the available concentrations of Cd, Pb, Zn, Cu and As in the As-Metals polluted soil (AM). For each column, different letters in different samples mean significant differences (n = 3, ANOVA; P < 0.05). Error bars represent standard deviation. Under limit detection (u.l).

The differences between As and metals availability in the soil AM are shown in Fig. 4 (P < 0.05). Pb, Cd and Zn showed a similar behavior. Specifically, Pb immobilization was remarkable and significantly increased as the doses of nGOx and nZVI rose (Fig. 4A,D, P < 0.05). However, Cd availability was only slightly reduced at the highest nGOx dose, whereas Zn availability presented a significant reduction at only the highest doses of nGOx and nZVI (Fig. 4B,E, P < 0.05).

In contrast, analysis of Cu and As availability revealed notable differences between nGOx and nZVI yields (Fig. 4C,F, P < 0.05). For Cu, significant decreases were detected with increasing doses of nGOx (Fig. 4C). However, nZVI treatment did not affect Cu availability, except for a slight increase observed at the highest dose.

Regarding As, opposite effects were observed, i.e., As availability was significantly increased as the dose of nGOx rose (Fig. 4C) while, on the other hand, it was clearly diminished after nZVI addition, more notably as the dose increased (Fig. 4F).

### Influence of nZVI and nGOx on the concentrations of available As in the As-polluted soil (A)

**Figure 5:**
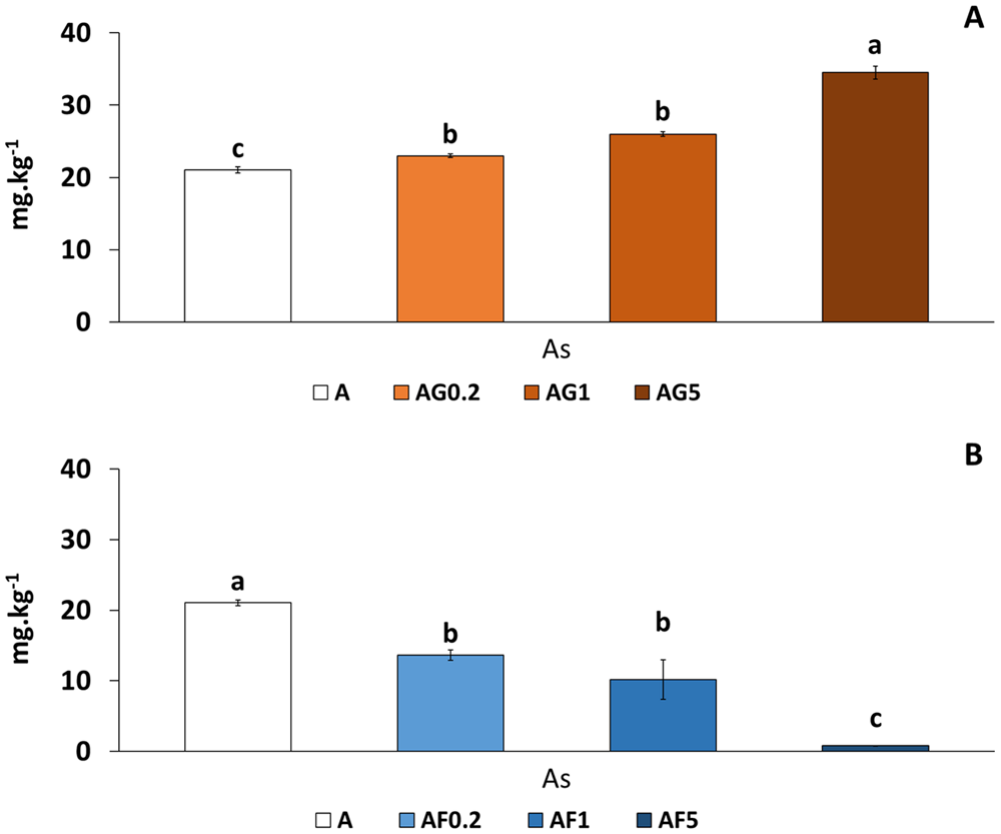
Influence of nZVI (**A**) and nGOx (**B**) on the available concentrations of As in the As-polluted soil (**A**). For each column, different letters in different samples indicate significant differences (n = 3, ANOVA; P < 0.05). Error bars represent standard deviation.

As explained above, and unlike soil AM, soil A presented only As pollution. Thus, Fig. 5 shows only variation in As availability in the different treatments. Regarding nGOx, the available content of As was increased as the dose of the nanoparticles rose, this increase being particularly marked at the highest dose (64% increase in As availability) (Fig. 5A). With respect to nZVI (Fig. 5B), the available content of As was reduced for all the doses tested, showing the greatest reduction at the highest dose (close to cero).

### Effect of nanocompounds on soil pH and electrical conductivity

Given the acidic (nGOx) and basic (nZVI) nature of the different compounds used, their effects at the studied range of dosages on soil pH and EC were examined. Soil pH has been slightly affected by nGOx being decreased as the doses increased in both soils, peaking at around 1.5 units of difference at the highest dose in the AM soil (Supplementary Table S1). On the other hand, nZVI has not affected soil pH. Regarding electrical conductivity, both types of nanocompounds increased it, more markedly for nGOx. However, electrical conductivity, even in samples at the highest doses, was lower than 2 dS.m^−1^, which strongly suggests that no salinity problems are promoted.

### Available phosphorus

**Figure 6:**
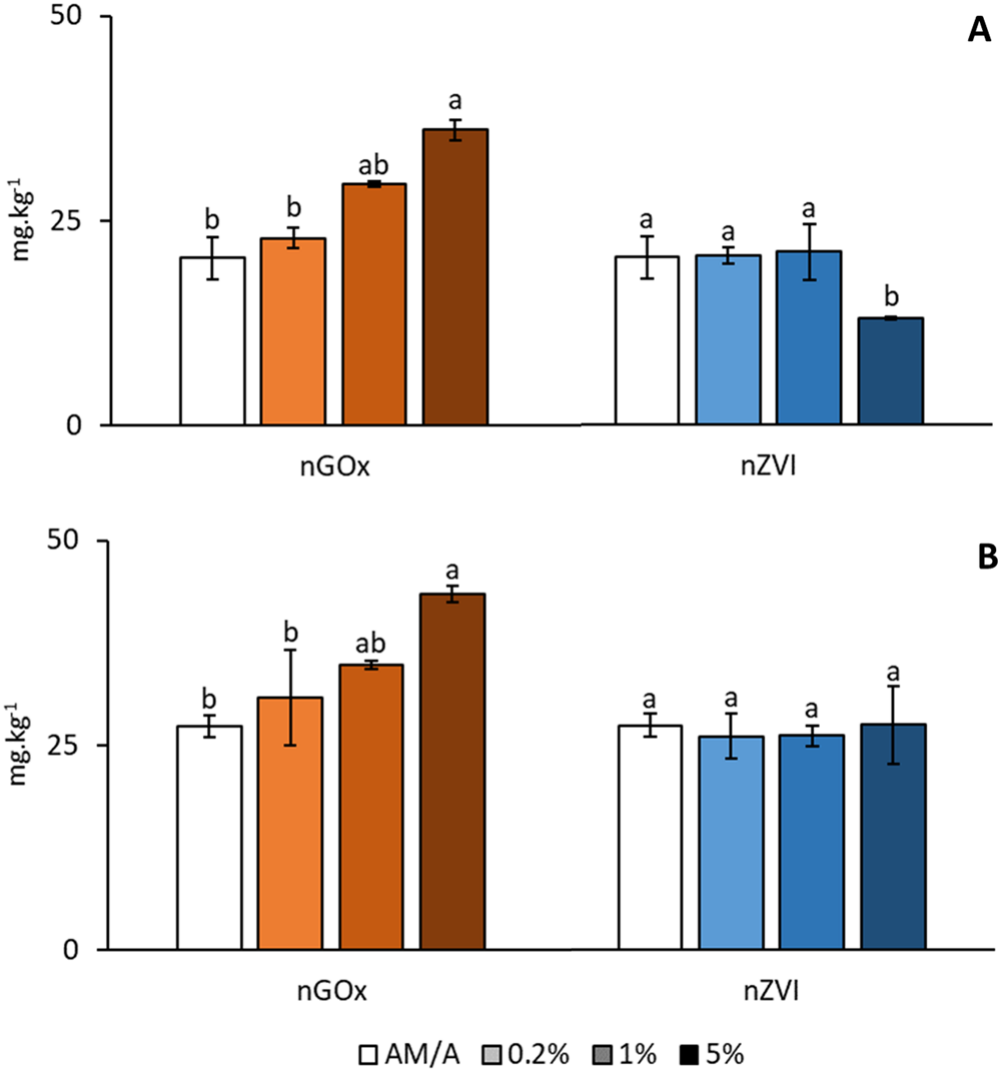
Influence on available phosphorus concentration by means of nZVI and nGO in the As-Metals polluted soil - AM- (**A**) and the As-polluted soil -A- (**B**). For each column, different letters in different samples indicate significant differences (n = 3, ANOVA; P < 0.05). Error bars represent standard deviation.

The response of available phosphorus (P) has been explored due to the foreseen competition between this parameter and As availability. Initially, P concentration in soil AM is lower than in soil A (Fig. 6). Respect to nGOx, the same effect on P was revealed in both soils: increasing doses imply an increase in P concentration (Fig. 6). This phenomenon is in accordance with the As behavior described above. In turn, nZVI treatment did not affected P concentration in soil A, and provoked a decrease at the highest dose in soil AM (Fig. 6).

## Discussion

### Arsenic and phosphorus availability

The results shown above for As were coherent for both soils (soil AM with accompanying high levels of metals, and soil A with As as the sole pollutant). In fact, the addition of nGOx caused an increase in As availability in both cases, which contrasts with the behavior observed for the cationic elements, as discussed below. The increase in available concentrations of As was positively correlated with the increase in nGOx dose in both soils (0.95; P < 0.01). In this regard, we hypothesize that the higher As availability in response to the addition of nGOx is due to the large number of oxygenous groups (COOH and OH) present on the negatively charged surface of nGOx, which is consistent with the potential zeta measurement referred to above. The increase of negatively charged organic carbon due to nGOx addition enhance desorption of As via competition^37^, thus the competition can occur between organic carbon and As for retention sites on soil^38^. Therefore, the electrostatic interactions between the negatively charged nGOx surface and the negatively charged As molecules (oxy anions of As^5+^) impede As sorption^39^. Conversely, in the nZVI experiments, the decrease in available As concentration was negatively correlated with increasing doses of nanoparticles for both soils (−0.89, P < 0.01); i.e., the effect of nZVI on As availability in this work (a notable decrease) is coherent with all the previous studies done with nZVI in As-polluted soils^11,40–42^. Consequently, these findings confirm the effectiveness of Fe° in stabilizing As in contaminated soils, and As is immobilized by nZVI via inner-sphere surface complexation onto iron oxides in the shell surrounding the nanoparticle^11,40^. These results have remarkable implications for future remediation approaches at pilot or real scale addressing polluted soils. nZVI are, as previously reported^40^, a good option to stabilize As and hence to be combined with phytostabilization or other immobilization processes^10^. In contrast, on the basis of our findings, nGOx emerge as a potential additive that can increase As mobility. This capacity opens up the possibility to enhance phytoextraction strategies in As-polluted soils by means of plant species such as *Pteris vittata*, *Brassica juncea, Helianthus annuus* or *Zea mays*^43,44^. From an economic point of view, the process should be addressed taking into account the cost of nGOx. The synthesis of this nanomaterial at large scale is still under development, and the market price is around 40€ per gram of nGOx in Europe. However, several studies are trying to reduce the manufacture costs, and the combination with a low cost technique such as phytoremediation could be economically viable in the medium term.

In soils A and AM, the increase in available concentrations of As caused by nGOx was due on one hand for the reasons explained above concerning the As-nGOx interaction, and on the other hand by a secondary effect affecting P concentration. The chemical behavior of As and P is similar because some of the forms in which As and P are found in nature are chemically and structurally analogous^45,46^. This was corroborated by the correlation between As and P in both soils (0.9, P < 0.01). Thus, the use of nGOx also increased the available concentrations of P in the soil due to the increase of organic carbon, similarly to As behavior. This increase in the P concentration in the soil facilitates As partial “desorption”, thereby increasing its bioavailability^47,48^. This is evident in this study given that the increase of nGOx doses implied an increase of the available P concentration and, in turn, that of As.

Respect to pH influence, which has been revealed as a key factor in As mobilization close to organic carbon and P concentration^49^, the nZVI addition did not affect this parameter whereas nGOx application slightly decreased pH. The soil pH is in both soils in a range between 7 and 8.5 while As is a chemically “mobile” metalloid within the pH range 6.5 to 8.5 both under oxidizing and reducing conditions^50^. Therefore, this slightly variation could favor As mobilization.

Once nZVI were applied in soils A and AM, and as previously mentioned, the available As concentration decreased in both cases while the available concentrations of P remained at similar values, with the exception of the highest dose in soil AM. In soil A, at the highest dose, the available As concentration was close to zero, so the surface area of the nanoparticles reacted with As. However, in case of soil AM, at the highest dose, available As concentration was zero. In that case, once As is fully immobilized, P could be able to react with the remaining nZVI thereby promoting the decrease of this value at the highest dose. This fact suggested that As was preferably sorbed by nZVI than P.

On the whole, it was demonstrated that nZVI and nGOx have a totally different behavior in their interaction with As.

### Metals availability

Regarding metals, the results obtained for soil AM are also relevant. In this context, nGOx reduced the available concentrations of Cu, Cd and Pb, although only at high doses for Cd. In fact, the decrease in the available concentrations of Cu, Cd and Pb was negatively correlated with the increase in nGOx doses (−0.98, −0.87, −0.978; P < 0.01). We propose that the functional groups of nGOx induce a negative charge on their surface, as verified by the zeta potential measure, and the oxygen atoms of these functional groups donate their only pair of electrons to the metal ions. This consequently increases the capacity of cation exchange of nGOx, thereby facilitating a decrease in the available concentrations of metal ions, with the exception of Zn (see below). This mechanism has already been observed in carbon nanotubes^21^. With regard to nZVI, the excellent results obtained in the immobilization of Pb are consistent with the findings of other studies^28,51,52^. This decrease in the available concentrations of Pb, and only slightly of Zn, negatively correlated with the increase in nZVI dose (−0.97, P < 0.01; −0.71, P < 0.05 respectively). In addition, our result coincides with the conclusions drawn by Gil-Díaz *et al*.^15^, who observed a higher immobilization effect for Pb than for Zn under the same conditions. Due to the high pH of nZVI, when these nanoparticles come into contact with the soil, they become excellent electron donors, thereby reducing Pb^2+^ ions to Pb^0^ or other insoluble forms^53^. Zn has a more negative reduction potential (−0.76 V) than Fe^0^ (−0.40 V) and is therefore immobilized by adsorption. However, in our case, the decrease in available Zn concentrations was modest. This may be attributable to saturation of the nanoparticles’ binding sites. This saturation may be due, on the one hand, to the fact that nZVI have a preference for adsorbing metals such as Pb, and on the other hand, to high available concentrations of Zn (33% of Zn in soil AM was in an available form). This differential behavior between Pb and Zn immobilization was also detected for nGOx, as indicated above, and it also occurs with other organic soil amendments, such as compost and biochar, where Zn is the metal that has the least affinity in the sorption sites of the soil^54^. In the case of Cd, the results for nZVI addition were negligible, as the standard potential was lower (−0.44 V) but very close to that of Fe (−0.40 V), and thus the mechanism of immobilization is not expected to involve reduction^55^. The lack of sorption effect of nZVI may be attributed to their preferential adsorption of As and Pb before Cd. In this regard, soil AM presented much higher available concentrations of As and especially Pb than those of Cd. This notion coincides with the conclusions of a study carried out by Li *et al*.^56^, who reported that the presence of As or Pb markedly decreased the capacity of Langmuir adsorption of Cd^2+^, thereby indicating competition for the active binding sites between As, Pb and Cd.

Unexpectedly, the application of nZVI at high doses caused an increase in the available concentrations of Cu (the effect of nGOx on Cu was the opposite). This increase in the available Cu concentration was positively correlated with the increase in nZVI dose (0.93, P < 0.01). This behavior is consistent with the results described by Hartley *et al*.^57^ and can be explained by the initial Pb concentrations being higher than those of Cu. As previously stated, in general, Pb is preferably adsorbed in comparison with Cu^54,58,59^. The behavior of Cu can also be explained by the alkaline pH of soil AM (8.31), which was enhanced by the addition of nZVI (pH = 11.5). According to Kumpiene *et al*.^41^, the mobility of Cu is usually low at slightly alkaline pH but can be increased in more alkaline conditions due to the formation of OH^−^ complexes.

On the whole, our results reveal the marked potential of nGOx to immobilize the metals of interest studied here. In this regard, nGOx outperformed nZVI (especially for Cu).

## Conclusion

The application of nZVI and nGOx to the polluted soils under study had different effects on the availability of As and metals.

Irrespective of the type of soil used, As availability was notably reduced when nZVI was applied and, conversely, increased with nGOx application. The differential effects observed between nZVI and nGOx introduce versatile options to immobilize As in stabilization approaches or to mobilize As in (phyto)extraction strategies. Furthermore, when using nGOx, the improvement of physico-chemical soil properties (for instance available P), also opens up the possibilities of applying hybrid approaches of phytoremediation and nanoremediation. In turn, the effects of nGOx on Cd, Pb and Zn were similar to that of nZVI, whereas the effectiveness of nGOx to immobilize Cu was significantly higher than that of nZVI, which generated an increase in Cu availability. On the basis of these observations, nGOx emerges as a suitable amendment to immobilize Cu in polluted soils.

Although the detailed mechanisms of the interaction between nGOx and the soil components were beyond the scope of this work, and should be clarified in future research, here we demonstrate the potential of nGOx as a complement or alternative to nZVI in nanoremediation technologies.

## Materials and Methods

### Samples and soil characterization

Bulk soil samples were collected from the surface layer (0–30 cm depth) of two polluted sites in Spain. The first soil used (soil AM) was sampled in an industrial area of the shoreline close to the city of Avilés, northern Spain, in the surroundings of a Zn smelter. Previous information about the site suggested potential Zn, Pb and As pollution^60^. The other soil (soil A) was taken from an abandoned industrial site in southern Spain, where characterization studies revealed As contents well-above soil screening levels^61^.

The physico-chemical properties of the two types of soil (Supplementary Table S2) were determined using the Spanish official methodology^62^. Samples were air-dried and sieved (<500 µm) prior to analysis. Soils were sieved <500 μm given that this soil fraction was the most polluted one in both soils. In this sense, the fraction 500–2000 μm was not considered in order to simulate real-scale conditions, in which coarse fractions with low contents of pollutants usually are separated before treatment to reduce costs. Organic matter was determined using the Walkley-Black method based on the dichromate oxidation principle. pH and EC were measured in a 1:2.5 soil-to-water ratio. The total nitrogen content was quantified by the Kjeldahl method. The percentage of carbonates was measured using a Bernard calcimeter, and available nutrients (Ca, K, Mg, Na) were extracted with 0.1N ammonium acetate and quantified using a flame atomic absorption spectrometer (FAAS). Available phosphorus (P) concentration was determined by Olsen method. Grain-size characterisation was carried out using the Bouyoucos assay. The total metal(loid) concentrations in the soil samples were determined after acid digestion with a mixture of 6 mL nitric acid (69% purity) and 2 mL of hydrochlorhydric acid (37% purity), in a microwave reaction system (Anton Paar). In this digestion extract, the concentrations of As, Cd, Cr, Cu, Hg, Ni, Pb and Zn were quantified by inductively coupled plasma mass spectrometry (ICP-MS, 7700 Agilent Technologies equipment) using IDA (Isotopic Dilution Analysis). Analyses were performed by duplicate. Detection limit for all elements is 0.1 µg.l^−1^, excepting for Cu, Ni and Zn, which is 0.25 µg.l^−1^. Available As and metals was determined by ICP-MS after TCLP extraction (Toxicity Characteristics Leaching Procedure) following the USEPA Method 1311^63^. Additionally, TCLP solution blanks were also measured in order to check the quality of the reagents.

### Synthesis and characterization of graphene oxide

The Hummers-Offeman method^64^ was used to synthesize graphene oxide. The method consists of the oxidation of graphite powder. In this case, a commercial micronized graphite powder (Sigma-Aldrich) was subjected to strong oxidation with KMnO_4_ in an acidic medium of H_2_SO_4_. A dark brown liquid was obtained after completion of the reaction. The graphene oxide powder was prepared by centrifugation of the solution and subsequent neutralization and washing. The suspension obtained was delaminated by redispersion of the powder in ultrapure water (conductivity below 5 µS.cm^−1^). The solution was submitted to treatment in an ultrasonic bath at a frequency of 40 kHz for 1 h.

Several analyses were conducted to characterize nGOx. The morphology and microstructure of the nanoparticles were studied by scanning electron microscopy (SEM) using a JEOL JSM-6400 Scanning Electron Microscope. The thickness of the nanoparticles was measured by atomic force microscopy (AFM) by means of Multimode Nanoscope III A (Bruker). For this purpose, samples were diluted in distilled water at three doses, 10, 1 and 0.1 mg.kg^−1^, and sonicated for 1 h. Subsequently, 10 µl of each suspension was deposited on a sample carrier of exfoliated mica and dried at room temperature. The elemental composition was determined using a LECO CHNS-932 analyser. Thermogravimetric analysis (TGA) was carried out to confirm the degree of oxidation of the particles. To this end, the particles were heated starting at 293 K and reaching 1073 K at the rate of 274 K min^−1^ under N_2_ atmosphere. In addition, the degree of oxidation was confirmed by powder x-ray diffraction (PXRD) of graphite and graphene oxide, on a Panalytical X-pert PRO Theta/2Theta. Finally, the specific surface area of the nanoparticles was determined by N_2_ adsorption at 77 K using an ASAP 2020 Micromeritics analyser. Samples were outgassed at 373 K for 2 h prior to the measurement. In addition, zeta potential of the nanoparticles were determined by dynamic light scattering (DLS) using a Zetasizer Nano ZS (Malvern Panalytical).

To determine the water suspension pH of graphene oxide, several solutions were prepared in deionized water (0.2%, 1% and 5% w/w) and submitted to treatment in an ultrasonic bath at a frequency of 40 kHz for 1 h. The pH of the resulting suspensions was measured using a Multiparametric HI 5521 (Hanna Instruments).

### Zero Valent Iron nanoparticles

The NANOFER 25S slurry is an aqueous dispersion of stabilized nZVI. According to the commercial specifications, the Fe^0^ content is 14–18%, and it also contains 2–6% of magnetite. The average size of the nanoparticles is close to 60 nm, the suspension is strongly alkaline (pH 11–12), and the active surface area is 20 m^2^.g^−1^ (additional details are available at www.nanoiron.cz).

### Batch experiments and monitoring

To test the effectiveness of nanoparticles to immobilize metal(oid)s, subsamples of 15 g of polluted soil were treated in vials with nZVI and nGOx at a range of doses. For nZVI, the doses chosen were based on previous experiments using this material (0.2%, 1% and 5% w/w) to treat other As-polluted soils^11,15,36,40,65,66^. The same doses were used for nGOx in order to facilitate the comparison of results. These doses (see Table 1) allowed comparison of the effectiveness of the nanomaterials across a broad spectrum (less, medium and high doses). Deionized water was added to reach the water holding capacity, and control tests were done only with the deionized water. Consequently, vials were shaken for 72 h at 100 rpm. After shaking, samples were air-dried. Experiments were performed in triplicate.

**Table 1:** Doses used to prepare the treatments.

Soil	Nanoparticles	Dose (%)	Code
**AM**	nZVI	0.2	AMF0.2
		1	AMF1
		5	AMF5
	nGOx	0.2	AMG0.2
		1	AMG1
		5	AMG5
**A**	nZVI	0.2	AF0.2
		1	AF1
		5	AF5
	nGOx	0.2	AG0.2
		1	AG1
		5	AG5

Following batch experiments, in order to evaluate the potential decrease of metal(oid)s leachability, TCLP extraction was performed over the three replicates. Additionally, soil EC and pH was also measured to reveal the impact of nanomaterials application, which is relevant due to the extremely pH of both types of nanoparticles. P concentration was also determined by Olsen method.

### Statistical analysis

All analytical determinations were performed in triplicate. The data obtained were statistically treated using version 24.0 of the SPSS programme for Windows. Analysis of variance (ANOVA) and test of homogeneity of variance were performed. In the case of homogeneity, a post hoc least significant difference (LSD) test was carried out. If there was no homogeneity, Dunnett’s T3 test was performed.

## Supplementary information

Supplementary material.
