# Supplementary material for: Nanoremediation of As and metals polluted soils by means of graphene oxide nanoparticles
Source: Sci Rep. 2020 Feb 5;10:1896. doi: 10.1038/s41598-020-58852-4 (PMC7002751; doi:10.1038/s41598-020-58852-4)
Supplement: Supplementary file 1 — Supplementary material. [file 41598_2020_58852_MOESM1_ESM.pdf]

Supplementary material

Nanoremediation of As and metals polluted soils by means of graphene oxide nanoparticles

Diego Baragaño[a*], Rubén Forján[a], Lorena Welte[b], J.R Gallego[a]

[a] INDUROT and Environmental Technology, Biotechnology and Geochemistry Group, Campus de Mieres, Universidad de Oviedo, Mieres (Asturias, Spain).

[b] Kleinscale S.L., Calle Montoro 4 9D, 28922, Alcorcón, Madrid, Spain

*Corresponding author E-mail: diegobcoto@uniovi.es

Supplementary Table S1. Mean values and error of pH and electrical conductivity of soil samples treated with nGO and nZVI nanoparticles. For each type of nanoparticles, data with the same letter do not differ significantly ($p<0.05$).

	Treatment	Dose	pH	EC ($dS.m^{-1}$)
**Soil AM**	Control	0%	8.46±0.04a	0.36±0.01c
	nGO	0.20%	8.23±0.05a	0.39±0.01c
		1%	7.75±0.04b	0.56±0.02b
		5%	7.18±0.03c	1.00±0.01a
	nZVI	0.20%	8.34±0.05a	0.40±0.01c
		1%	8.27±0.04a	0.45±0.01b
		5%	8.40±0.04a	0.62±0.01a
**Soil A**	Control	0%	8.54±0.03a	0.17±0.01c
	nGO	0.20%	8.32±0.03b	0.28±0.01b
		1%	8.14±0.01c	0.34±0.01b
		5%	7.84±0.04d	1.12±0.05a
	nZVI	0.20%	8.52±0.03a	0.20±0.01c
		1%	8.52±0.03a	0.19±0.01c
		5%	8.51±0.01a	0.28±0.01a

Supplementary Table S2. Soil AM (polluted by As + heavy metal) and soil A (polluted by As).

Parameters		Units	Soils	
			AM	A
General	pH		8.31	8.91
	OM	%	1.54	0.2
	TN	g·kg^{-1}	0.6	0.2
	Available-P	mg·kg^{-1}	22	27
	K		1.6	3.8
	Mg		7.1	20.6
	Ca		18.2	14
	Na		3.5	1.1
	Silt	%	6.91	3.81
	Sand		87.9	80.7
	Clay		5.21	15.5
Pseudototal Concentration	Ni	mg·kg^{-1}	8.41	23.1
	Cu		79.0	19.1
	Zn		4,900	47.1
	As		37.2	1100
	Cd		17.1	<DL
	Pb		390	14.1
Available Concentration	Ni	mg·kg^{-1}	0.30	<DL
	Cu		2.77	<DL
	Zn		1,635	0.32
	As		0.91	21.0
	Cd		6.77	<DL
	Pb		21.0	<DL

Values (n = 3). <DL: Below detection limit; OM: organic matter; TN: total nitrogen; Bioavailable-P= available phosphorus (Olsen Phosphorus).
